# Preparing for parenthood: developing a life-skills and socioemotional health program for young married couples in rural Pakistan

**DOI:** 10.1080/16549716.2021.1982485

**Published:** 2021-10-04

**Authors:** Najia Atif, Atif Rahman, Zill-e- Huma, Syed Usman Hamdani

**Affiliations:** a Child and Adolescent Mental Health, Human Development Research Foundation, Rawalpindi, Pakistan; b Department of Primary Care and Mental Health, University of Liverpool, Liverpool, UK

**Keywords:** Life-skills, adolescents, sexual and reproductive health, mental health, low resource settings

## Abstract

**Background:**

Socioemotional life-skills to negotiate important life-transitions such as marriage and parenthood are critical for the wellbeing of young couples and their offspring, but programs addressing this issue are lacking in Low and Middle-Income Countries (LMICs).

**Objective:**

This study describes the development of a ‘life-skills’ program for young married women, their husbands and families, living in rural settings in Pakistan.

**Methods:**

Our methods included: a) a targeted review of relevant literature on life-skills and mental health in young people, b) a qualitative study and, c) intervention development workshops with experts and stakeholders. The review showed that common life-skills employed as part of psychosocial interventions in LMICs were communication skills, problem-solving, assessing relations, stress management, emotional regulation, identifying/eliciting affect, and self-awareness.

**Results:**

The qualitative study indicated that areas of particular need included interpersonal communication skills with significant others, coping with the pressures of parenthood, and mental well-being. Existing helpful practices included social support by family members and elders. Lack of empowerment in young married women and poor engagement of husbands were identified as a barrier to accessing a potential intervention. Our proposed intervention called ‘Preparing for Parenthood’ consisted of 10 core sessions and 10 follow-up sessions designed to be delivered by lay health workers. It synergistically combined evidence-based socioemotional life-skills (awareness, communication skills, assertiveness, decision-making skills, goal-setting, critical thinking, problem-solving, and coping with stress), with cognitive behavioural strategies (gently challenging existing thoughts and attitudes, behaviour activation and problem solving). The intervention focuses on engagement of the entire family, including husbands.

**Conclusions:**

The intervention can supplement existing sexual and reproductive health programs by providing skills to prospective parents to effectively negotiate stressful life-transitions and life-events. We envisage the intervention would improve mental as well as sexual and reproductive health of young couples and plan to test this in future randomised trials.

## Background

Young people aged 15–24 years represent 16% of the world’s population or around one in every six persons worldwide [1]. The socioemotional health of young people has largely been neglected [2] especially in Low and Middle-Income Countries (LMICs), despite its proven adverse effects throughout life [3]. In Pakistan, the number of young people aged between 15 and 24 years is 44 million, which account for nearly 21% percent of its population. Marriage at a young age is common-practice and disproportionately affects women from rural and lower socioeconomic backgrounds. According to the Pakistan Demographic and Health Survey (2017–2018), 29% and 47% of women and 5% and 14% of men were married by the age of 18 and 20 respectively [4]. The birth rate in Pakistan is also amongst the highest in the world with 10% of women giving birth before the age of 18 years [5]. Young couple are often unprepared for the transition to parenthood that involves many life events including role changes, childbirth and increased financial pressures [6]. If not prepared for, these transitions can have negative effects on socioemotional health. In one rural area of Pakistan, we found a five-fold increase in the prevalence rates of depression among women aged 20 years old or less following marriage and during pregnancy, as compared to unmarried women aged 18 [6]. Several potentially difficult life events clustered together such as interpersonal problems, increased household responsibilities, pregnancy, and childbirth were found to contribute to this. Lack of autonomous decision-making in women coupled with limited access to sexual and reproductive health information adds to the challenges of parenthood [7]. According to the Pakistan Demographic and Health Survey (PDHS) 2017–2018, 17% of currently married women have an unmet need for family planning. Furthermore, only 25% of currently married women age 15–49 use modern contraceptives and its usage is lowest (7% and 18%) among younger women (aged 15–19 and age 20–24 respectively) [4]. Consequently, young women experience high fertility, rapid repeat pregnancy and unwanted pregnancy [8]. In Pakistan, the most common cause of abortion is unwanted pregnancies, resulting due to unmet need for family planning or contraceptive failure, and the lack of the legal and safe abortion services results in high morbidity and mortality associated with unsafe abortion [9–11].

The above evidence suggests a need to develop and test culturally appropriate ‘life-skills’ interventions in such settings aimed to help young married couples negotiate life transitions, deal effectively with post-marriage challenges, and make appropriate decisions about their physical, psychosocial, sexual and reproductive health. Such interventions need also to focus on the socioemotional health of the couple, especially young women. This study describes the development of a ‘life-skills’ program for young married women aged between 18 and 22, their husbands and families, living in poor rural settings in Pakistan.

## Method

### Research design

Our intervention development process was conducted in three stages: a) review of relevant literature; b) qualitative study to explore psychosocial problems faced by young married couples, existing practices used to deal with such problems, and possible facilitators and barriers in the delivery of such an intervention in the community; c) intervention development workshops with experts and stakeholders.

Our approach is informed by the UK Medical Research Council framework for the development of complex interventions [12], which recognizes iterative phases of i) Intervention Development; ii) Feasibility and Piloting; iii) Evaluation; and iv) Implementation. The present paper describes the intervention development process which consisted of three stages; a) review of relevant literature; b) qualitative study; c) intervention development workshops with experts and stakeholders.

The aim of the literature review was to inform the development and implementation of life skills program for adolescents in LMICs; it involved describing and evaluating life skills components to improve mental health outcomes in adolescents and identifying other complementary participatory approaches and psychological therapies including Cognitive Behavioral Therapy (CBT). The qualitative study aimed to identify the priority problems faced by the young couples; understand the perceived causes and impact of the psychosocial problems on them; explore current practices and potential mitigation strategies in addressing these problems. The qualitative study also explored the feasibility and acceptability of delivering such an intervention in the community.

### Review of literature

A systematic review focusing on the effectiveness of life skills programs among adolescents in LMICs was conducted [13]. The detailed methodology and findings are presented elsewhere [13], but we systematically searched six academic databases, including PubMed and PsychInfo and bibliographies of related reviews, with no restrictions on language or publication year. Only randomised controlled trials conducted in low- and middle-income countries were included. Data from published reports related to the characteristics of RCTs and their implementation processes related to ‘who, what, how and where’ were extracted, including the development of a taxonomy to determine which life skills constituted each program. We also conducted a desk review of relevant articles and reports on this subject using Google Scholar and PubMed. Publications and manuals that had relevant materials on this subject matter were reviewed. Abstracts/papers and documents were considered if they had information that was relevant to our objectives and published between 2017 and 2020. The purpose of the review was to appraise what evidence there was for content and structure of life skills interventions, their overlap with other mental health interventional approaches such as Cognitive Behaviour Therapy (CBT), and existing intervention packages that incorporated these evidence-based practices.

### Qualitative study

#### Aims and objectives

The qualitative study was aimed to understand the perceived causes and impact of the problems experienced by the young couples, current practices in addressing these problems and the likely barriers and facilitators for the delivery of the Life Skills Programme to the adolescent couples in our settings.

Our specific research questions were aimed to explore; the priority problems faced by young married women, their husbands and families, living in poor rural settings in Pakistan, the perceived causes, psychosocial impact of these problems, current practices and proposed mitigation strategies to address these problems, as reported by young married women, their husbands and families, living in poor rural settings in Pakistan.

##### Settings and participants

The study was conducted in May 2018 to January 2019 in the Union Council of Kallar Syedan, a rural sub-district of Rawalpindi, located in the province of Punjab, Pakistan. It has an estimated population of 300,000. The economy is primarily agrarian-based, and majority of families live in a joint family system (multiple generations living in the same house) with an average of 6.2 persons per household. The study area has high rates of poverty (up to 25% living on less than US$3.5 per day), high fertility rates (3.8 birth per woman) and low levels of female literacy (less than 45% literate). The society is largely patriarchal, with women generally economically and socially dependent on male members of the family. About 95% of the women in the study area are registered with the Community Health Workers (CHWs). The CHWs were asked to purposively identify women from their catchment area who were aged between 18 years to 22 years. A smaller sample of significant family members and local CHWs were also invited. Interested participants were approached by the trained research team, and upon receiving their informed consent interviews were conducted.

##### Sample

In total, 25 interviews were conducted. Participants [not necessarily part of the same family] included; 1) men, married for less than 5 years (n = 4) and more than 5 years (n = 1), 2) women married for less than 5 years (n = 9) and more than 5 years (n = 3), 3) mothers-in-law (n = 2) and 4) CHWs (n = 6). Women’s ages ranged from 18 to 22 years and men’s ages ranged from 23 to 28 years, and years of schooling for both ranged from 8 to 14 years. Majority of the participants were living in the joint family system (consisting of multiple generations; parents, their children, and the children’s spouses and offspring, living in the same house).

##### Procedures

Data were collected through in-depth interviews. Topic guides were developed and pilot tested. The key areas of the topic guide included; understanding the perceived psychosocial problems and perceived causes and impact of the psychosocial problems experienced by young couples, current practices in addressing these problems and the likely barriers and facilitators for the delivery of an intervention program in the community. Interviews were conducted by trained researchers and recorded following permission from the participants. Each interview lasted between 30 to 60 minutes. All interviews were transcribed and analysed.

##### Data analysis

Thematic Analysis was applied to analyse the data. The data was analysed by the lead author (NA) and 3 Research Assistants trained in the qualitative methods. The transcribed data was familiarized by reading and rereading, followed by manual coding and code sorting to form sub-themes. Coders worked in pairs, to avoid bias in the analysis. Any discrepancies in the findings were discussed and resolved through discussion, revisiting the raw data and referring to the field notes. Similar sub-themes were clustered together to form themes. Themes were revised and finalised in relation to the data and were given names. Illustrative quotes were selected from each theme.

### Intervention development

Intervention development involved a systematic triangulation process by which findings from the literature review and our qualitative study were combined to obtain an in-depth understanding of the issues involved in designing and delivering the proposed intervention. An expert panel, comprising of the psychiatrists, psychologists, and local mental health researchers, reviewed the synthesised data to develop the content and select the life skills domains and delivery approach most suited to the population that could be adapted in line with conclusions drawn from the data.

## Ethical approval

Ethical approval for the study was obtained from the institutional review board of the Human Development Research Foundation (HDRF), Islamabad. All participants gave written informed consent before participation in the study.

## Results

### Review of literature

The United Nations Children’s Fund (UNICEF) broadly defines life-skills as ‘adaptive and positive behaviours that enable individuals to deal effectively with the demands and challenges of everyday life’ [14]. Evidence from the High-Income Countries (HIC) indicates that multicomponent interventions aiming to strengthen ‘life skills’ through addressing emotional regulation, self-efficacy and conflict resolution are effective [15]. Our systematic review identified 45 trials of psychosocial interventions targeting adolescents in LMICs which had at least one element derived from the area of ‘life-skills’ as defined above. A detailed description, including met-analyses of the studies is described elsewhere [13]. For the purposes of this study, our key findings were that in older adolescents, the most commonly employed life-skills were communication skills (57.8%); problem-solving (53.3%); assessing relations (51.1%); stress management (44.4%); emotional regulation (44.4%); identifying/eliciting affect (37.8%), and self-awareness (35.6%). The effectiveness of the psychosocial intervention in older adolescents was most strongly associated with life-skills in the area of interpersonal relations and stress management. Most of the interventions were delivered by non-specialists (such as teachers) and most were focused on high-risk groups rather than clinically disordered populations.

Review of related literature showed a number of life-skills programs addressing adolescent students’ sexual and reproductive health indicators [16]; building livelihoods skills [17]; changing prevailing youth norms [17]; addressing gender role attitudes [18]; and enhancing the agency of adolescent girls [19] showed promising results. Studies conducted in India and Nepal targeted young females and focused on their reproductive health, prevention of early pregnancies, birth spacing and postnatal care [20–22]. We found very little in terms of manualised interventions with a comprehensive theoretical framework that specifically targeted young married couples and was designed for LMIC. The Life Skills Education Toolkit (LSE toolkit) developed by World Health Organization for younger unmarried adolescents aged 12–16 attending school [23] provided a framework that focused on eight domains of life skills; self-awareness, communication skills, assertiveness, decision-making skills, goal-setting, critical thinking, problem-solving, and coping with stress. The LSE toolkit was aimed to give adolescents the knowledge, skills, values and attitudes through educational sessions that could help them make healthy lifestyle choices and become adaptive and productive members of their society. The domains and strategies in the WHO framework mapped on well to the domains and life-skills identified to be most effective in the our LMICs review [13].

### Qualitative study

Following analysis, five key themes were generated and are described below.

## Theme 1: interpersonal problems with significant others

### Interpersonal problems with in-laws

The majority of wives interviewed reported, multiple stresses related to adjusting to extended family members living in often overcrowded homes. They described the burden of expectations from their in-laws and pressure to keep everyone happy to gain acceptance to their new family.

After getting married, first problem is to deal with so many in-laws living in the same house, and the pressure to please everyone there (W09)

Terms used by the participants to describe the problems included ‘harsh attitudes’; ‘interference and restrictions’; judgemental attitude’; ‘undue criticism’; and ‘excessive work responsibilities’. The young married couple’s lack of autonomy in making decisions about their day-to-day life was frequently described by all the participants, as reflected in the quote from a mother-in-law below:

Husband and wife are not allowed to go out of home alone without permission. They have to ask for permission from mother in-law, sisters in-law etc. (MIL19)

There were a few contrary viewpoints, and largely voiced by the older CHWs. The main ones were that some young women showed lack of tolerance and patience towards their in-laws; they were preoccupied with ideas of living independently instead of being a part of the joint family; some took no interest in their domestic responsibilities which led to more work for other female family members, causing tensions.

Some girls demand a totally separate (independent) life, they don’t want to live in joint family system, and that causes friction between them and their in-laws (W08)

### Interpersonal problems with partner

The main sources of tensions between the married partners included: controlling and suspicious attitudes of the spouse, a lack of understanding between the couple; and differences between partners in their families’ socioeconomic status, education and age and marriages forced upon them by their families. A husband reported the consequences of a forced marriage by stating:

If couple is married against their will, then they will keep blaming and quarrelling with each other which leads to so many problems in their marriage (H16)

Husbands’ unemployment often led to reliance on the pooled family resources, creating tensions between the partners. Participants also reported interpersonal problems exacerbated by getting married at a younger age when they lacked the maturity to deal with marital responsibilities.

There are lots of problems if a girl is married at younger age; problems in understanding her husband, taking care of home and taking care of herself (MIL19).

Other issues affecting spousal relations included: in-law’s (especially mother-in-laws’) interference in the couples’ private affairs and sometimes even instigating sons against their wives; spouses’ extended families falling out with each other. The negative role of television and social media was mentioned by several participants, where serials portraying family members as villains or victims tended to exacerbate tensions:

Women watch liberal shows on mobile, Facebook and TV, this creates problems later on their relationship with their husbands (W15)

## Theme 2: pressures of parenthood

### Pressures of conceiving

Majority of the wives reported the pressure of getting pregnant soon after their marriage, with failure to do so resulting in accusations of infertility and even subtle threats of divorce. Men were exempted from blame, and in most cases, refused medical check-up, as this is perceived as a threat to their manhood.

If a woman is not conceiving, then her mother in-law will ask her son to leave his wife and get married to another woman (MIL19)

Participants described multiple pregnancies with little birth spacing affecting the health of women and their children. They reported religious stigma, especially from family elders, around the use of contraception, despite knowing the risks of multiple pregnancies such as miscarriages and difficult labour.

If there is no gap in-between child birth, three children are born within two to three years then she can experience poor health during pregnancies and will have trouble looking after her children (MIL19)

Another pressure experienced by women was to produce male off-spring. Giving birth to a female offspring was generally looked down upon and the woman would be blamed for it. This would often lead to repeated pregnancies in an effort to produce a male child.

If the woman doesn’t conceive, she is blamed for it, they (in-laws) will whisper in man’s ear to give her divorce, send her back (maternal) home and they will say to the woman you are barren if we knew you can’t have children we should not have married our son to you at the first place (W22)

This pressure was also endorsed by a husband who reported:

In-laws wants the first baby to be male child. If a mother fails to give birth to a male child, then in-laws will criticise her (H16)

### Pressure of becoming a parent

The participants reported that women were expected to provide care to their new-born alongside managing their domestic responsibilities. This was very challenging for the young inexperienced mother, especially if the other family members were not supportive.

Mother in-law does not tolerate if her daughter-in-law will sit idle (during post-natal period), she asks her to do housework with other daughters-in-law or else she can go back to her maternal home (MIL19)

Husbands also reported increased pressures on women if they are young and financially struggling and on men to provide for their growing family on becoming a father.

It is hard for a young mother to look after her children, it gets harder when her husband is not earning well, then she will have to face more difficulties in fulfilling needs of her children (H16)

Similar pressures were reported by a CHW during her interview.

As the family grows it becomes more and more difficult to make both ends meet. Wife starts demanding things, (such as) baby needs pampers, and the husband is not earning enough to fulfil her demands – it gets more and more difficult for the man to cope with it as the family grows (CHW12)

## Theme 3: impact of the problems identified

### Impact on personal wellbeing

The majority of the participants reported a significant impact of these problems on personal well-being, both physical and emotional. The key experience was psychosocial distress, which ranged from excessive worry, anxiety and depression to having suicidal thoughts.

I feel angry, thinking why did I get married, all this is happening because of my marriage. I often think I should do something, go away somewhere far, maybe die (W07)

Common physical complaints included hypertension, restlessness, weakness, anaemia, tiredness, aches and pains.

When someone suffers from mental stress blood pressure sometimes rises and other times it falls. (W14)

### Impact on family relationships

Most participants reported interpersonal problems leading to serious consequences, which could even lead to a complete breakdown of the marriage. Frequent arguments could escalate to separation, divorce or men taking on a second wife.

They argue, they fight and eventually they stop loving each other. It is easier for men because they can divorce their wives and marry again (W14).

If husband listens to his mother, then wife will say that he doesn’t listens to me and if he listens to his wife, his mother will say that he doesn’t obey me. No one in the family talks to each other except negatively. (MIL19)

### Impact on children

Parental stress often manifested in the form of shouting at or hitting children. This was especially the case with women who married early and were not prepared for the maternal role. Fathers would tend to remain absent from home and not engage with the children when there were psychosocial problems.

Due to pressures and quarrels (between wife and husband/in-laws), mother will scold her children. There will be problems in their upbringing, and it will impact their progression (CHW3)

## Theme 4: existing helpful practices

The key helpful practice hinged around social support, advice and practical help by family members and was reported by the majority of the participants. The most often cited sources included elders in the families, neighbours, spiritual leaders and community counsellors. In addition to giving support with practical concerns like domestic responsibilities and childcare, they often played the role of mediators, and in most tried to offer impartial advice to resolve marital differences.

There are different people in family who do marital mediation, people call them and they help sorting out their differences (M15)

Husbands also mentioned similar resources within the community, from which marriage counselling and guidance can be sought.

People go to the village counsellor and elders for advice. They are resourceful and help in getting their problems solved (H16)

The other commonly reported practice was ‘patience and forbearance’, often linked to faith, and practiced more by women than men. Many women felt they were obligated to compromise for the sake of their children or the social taboos associated with divorce.

Those who have patience, learn to bear and with time problems starts settling. First three to four years are tough for women after getting married after that things settle down (MIL18)

A compromising option was reported by a husband, who felt that joint families with complex interpersonal problems are better off living in separate homes:

Sometimes family separates two brothers. When there is lesser interaction between two families, chances of conflicts are less (H9)

## Theme 5: barriers and facilitators to a potential intervention

The biggest perceived barrier was women’s lack of empowerment. The Majority of the participants felt young married women would require their in-laws’ permission to receive such an intervention, and this might be refused due to fear of disclosure of family matters to an outsider and anticipating that receiving sessions will impinge on the time allocated to the domestic responsibilities.

In-laws will hesitation thinking that their daughter in-law will discuss family matters with the outsiders, which might bring shame to the family (H9)

The other key barrier was perceived to be the likely lack of engagement from the men because of their unwillingness to receive any interventions and/or to change their attitudes and behaviour. As stated by a young husband himself in his interview:

No matter how many programs you start, there are no use to men, as they are very stubborn, they are not willing to change or to receive any intervention (H21).

The main facilitator reported by the majority of the participants was linked to the importance of social and family support available to young people, especially from family elders. The participants recommended involving such family members in the intervention as this will would ensure the family’s support for the young couple and will enhance the acceptability of the intervention. One mother-in-law stated:

Involve everyone from home, then you have a better chance of delivering the program otherwise they will keep suspecting not knowing what is happing during the sessions or may not even allow you to come in (MIL19)

The place of intervention-delivery was also considered an important facilitator or barrier. Women were unlikely to attend if the intervention was delivered in a health facility and more likely to attend if it was delivered at home. It should be delivered by trained people and the sessions’ duration should not be longer than an hour.

If there are some people who are trained properly, they can go from home to home and deliver this program in an effective way (MIL18)

The topics of particular interest to the majority of the participants included raising awareness about reproductive health issues and strategies for coping with challenges of parenthood. The majority of the participants felt the interventions should help young couples to deal with marital responsibilities with mutual respect, support and taking care of each other’s needs. All participants agreed that the expected child and his or her health and optimal development was the shared agenda of the entire family. Thus, the child’s well-being could be the common entry point for engaging all key family members.

Those who got married at younger age they should be trained in how they can run a house, how they can be more tolerant, how to manage housework without getting pressurised. In your program you should tell young people how to improve their situation, tell the pregnant woman to take care of her health. Everyone should help the couple. It is for their future generation after all (CHW10).

### Designing the intervention

An expert panel, comprising of the psychiatrists, psychologists, and local mental health researchers, and potential users examined the data from the literature review and the qualitative study, and developed a manualised ‘life-skills’ intervention for young married couples, building on existing evidence-based elements that addressed the key problems raised in the qualitative study. The themes emerging from the qualitative interviews were appraised in the light of the findings of the literature review and the interventions that best matched the identified needs were incorporated into the new intervention. The intervention, called ‘Preparing for Parenthood’ had the following features:

## Key features of the intervention

### Addressing key problems through life-skills training

The 8 domains of life skills (awareness, communication skills, assertiveness, decision-making skills, goal-setting, critical thinking, problem-solving, and coping with stress to help making healthy life choices) identified in the WHO framework [14] were mapped on to the problems identified in our qualitative study (interpersonal relationships and pressures of parenthood). Using the technique of distillation and matching described by Chorpita and colleagues [24] we selected techniques described in the WHO Toolkit that were most likely to address the problems identified in our qualitative study. These included: awareness, communication skills, assertiveness, and decision-making skills.

### Addressing psychosocial well-being

Our qualitative study showed that stress, anxiety and depression in participants was a major theme that needed to be addressed. We used elements derived from the evidence-based Thinking Healthy Program [25,26] which used CBT principles to address psychosocial distress in the perinatal period. A feature of this well-tested intervention is that the key strategies can be integrated into other programs [26]. The key CBT strategies used in the Thinking Healthy Program included family engagement, thought challenging, behavioural activation, and problem solving. We integrated these strategies into our ‘Preparing for Parenthood’ life-skills program.

In this process, we identified overlaps between CBT strategies and the domains of life-skills based interventions. For example, critical thinking overlapped with thought challenging; goal setting with behavioural activation; and problem-solving was common to both. We employed the *common elements approach* to integrate these overlapping elements. The common elements approach is the concept of identifying specific techniques or components that overlap across multiple evidence-based interventions so that these common elements can be taken together and integrated as a synergistic intervention [24]. There are several advantages to this approach. There is greater flexibility for the health worker to tailor the intervention to the needs of the particular client and family. It is less confusing for mothers and families and avoids conflicting messages from multiple health workers working with overlapping objectives. But the greatest advantage is that it combines a number of overlapping theoretical approaches into a single intervention with synergistic effects across the range of domains.

### Addressing key barriers to intervention

Finding from the qualitative study indicated lack of endorsement from the family as a major barrier in acceptability, and family support as a key facilitator. This highlighted the significance of involving the family members during the intervention delivery. While the intervention targets young married couples and encourages them to attend the sessions together, it also promotes the participation of the significant family members such as parents-in-law. This would help address their concerns, ensure support and enhance acceptability of the intervention. Family members would become familiar with and even practice life-skills alongside the participants, which could facilitate positive outcomes for the entire household. We incorporated techniques that could engage not just the young couple but also key significant family members in the intervention. These included:

#### Use of narratives and stories

Narratives have great potential for engaging participants, stimulating insight, guiding listeners, challenging deeply embedded beliefs and practices and facilitating behaviour change [27]. We included culturally appropriate and relevant case scenarios developed in consultation with the local women to depict everyday different challenges and life situations. They aimed to help participants reflect and gain better insight into their own problems, think of alternative perspectives, share personal experiences and suggest ways of dealing with them. In addition, these case scenarios gently challenge their cultural norms, existing knowledge and attitudes.

#### Interactive learning approach

Interactive learning approach is a hands-on approach, where participants are invited to interact together and work in collaboration to achieve specific goals. To make the intervention more interactive, various tools such as vignettes, card games and group activities were developed. For example, each vignette ended with a set of interesting quizzes. Following narrating the vignette, the facilitator encouraged participants and their families to discuss the scenario and come up with the possible responses to the questions. This approach helped in improving participants’ engagement, cooperation, communication skills, decision-making skills, problem-solving and critical thinking.

### Conceptual framework of the intervention

**Figure 1.**
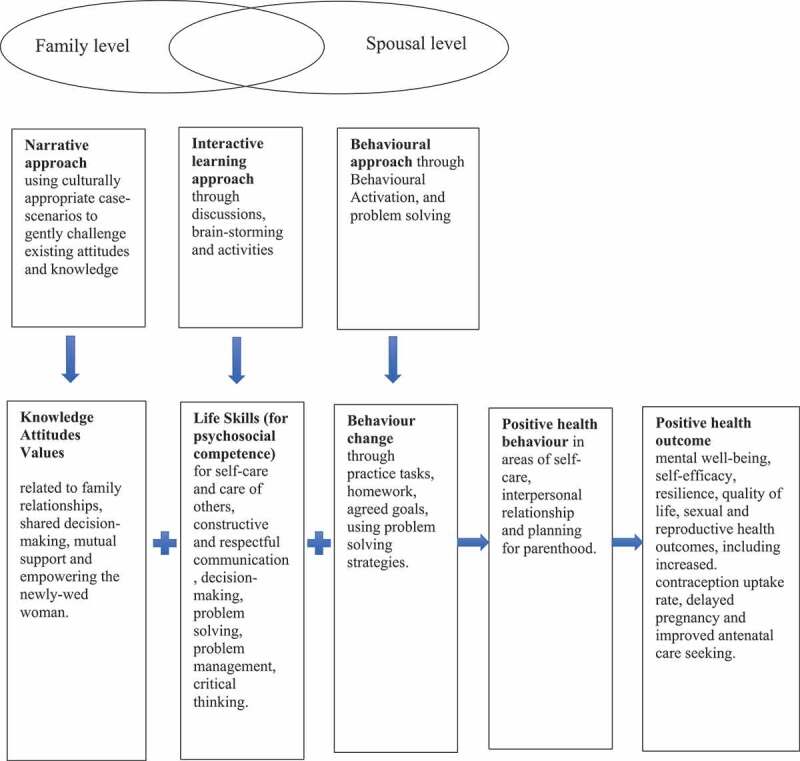
Conceptual framework of the intervention

Figure 1 illustrates the conceptual framework for the intervention. We hypothesized that synergistic employment of knowledge, attitude and values related to family and relationships, life-skills (to build psychosocial competence) and behaviour change component will lead to improvement in the three key problem areas (relationship with self, spouse and family member), which in turn will help achieve positive health outcomes such as mental well-being, self-efficacy, resilience, quality of life, sexual and reproductive health outcomes, including increased contraception uptake rate, delayed pregnancy and improved antenatal care seeking.

The proposed pathway to change included the following:

1. Gain in knowledge, raised awareness and changed attitudes related to personal wellbeing, family relationships, shared decision-making, mutual support and empowering the young married woman
2. Learning new skills for self-care and care of others, constructive and respectful communication, decision-making, problem solving, problem management, critical thinking.
3. Changed behaviour through practice tasks, homework, agreed goals, using problem solving strategies.

### Preparing for parenthood format and structure

Based on feedback from participants, the intervention was designed to be delivered at home in weekly sessions, with each session lasting no longer than an hour. The total duration of the intervention delivery was between 5 and 6 months. It comprised 20 sessions: 10 core and 10 follow-up sessions, delivered every week. In the core sessions, participants were encouraged to take part in specially designed activities, engage in discussions and brainstorming sessions to become familiar with life skills. The follow-up sessions reviewed the knowledge and skills and focused on discussing the participants’ experience of practising the life skills they learnt in the core sessions. The structure of each session and the targeted area are given in Table 1.

**Table 1.** Preparing for parenthood core sessions’ delivery schedule

Session 0: Introduction to the program	
*Core sessions 1 to 10	**Major focus of session**
Session 1: Knowing and appreciating each other	Family support; Communication skills
Session 2: Looking after yourself	Thought challenging; behavioural activation
Session 3: Looking after yourself and others around you	Thought challenging; behavioural activation
Session 4: Listening and understanding each other	Communication skills; awareness
Session 5: Communicating respectfully and positively	Communication skills: assertiveness
Session 6: Seeking and valuing each other’s opinion	Communication skills; decision making
Session 7: Preparing for parenthood: pre-pregnancy	Awareness and education of pre-pregnancy care; behavioural activation; problem solving
Session 8: Preparing for parenthood: during-pregnancy	Awareness and education of care during pregnancy; behavioural activation; problem solving
Session 9: Preparing for parenthood: post-pregnancy	Awareness and education of post pregnancy case; behavioural activation; problem solving
Session 10: Managing problems	Problem solving

* Each core session has a follow-up session, delivered the following week, making a total of 20 sessions.

#### Training and supervision

The intervention was designed to be delivered by lay health workers. They would receive 35 hours of classroom training delivered by the lead trainer. The training would focus on understanding the importance of life skills, counselling skills, approaches used in the intervention, the core topics and their delivery procedures, and use of monitoring protocols. Throughout the training, lay workers would be assessed on knowledge and skills required to deliver the intervention using role-playing exercises.

Following successful completion of the classroom training, the participants would receive field training during which they will deliver 10 core sessions to the couples similar to the target population. This phase of training was envisaged to provide first-hand experience of delivering the intervention in real-life settings under close supervision.

## Discussion

In this formative research, we examined the literature on life-skills interventions for young people in LMIC that might address the challenges associated with transition to parenthood. We studied the key problems faced by young married couples in a rural setting in Pakistan. Our key findings indicated that there was a dearth of interventions specifically addressing the issues of young married couples. Using the insights gained from our research, we developed a culturally acceptable intervention that addressed these issues and could potentially be delivered feasibly in such settings.

The key issues identified through our qualitative study related to interpersonal problems with significant family members, including the in-laws and spouse; the lack of preparedness to meet the challenges of becoming a parent, and psychosocial distress. Interpersonal problems are frequently described in the literature as a significant contributor to poor well-being, especially in women [28]. Such problems can escalate to domestic violence [29]. According to the Pakistan Demographic and Health Survey (2017–18) [4], 34% of ever-married women have experienced spousal physical, sexual, or emotional violence. The most common type of spousal violence was emotional violence (26%), followed by physical violence (23%). Smaller, clinic-based studies have produced even highly variable estimates ranging from 34% to 57.7% for lifetime physical violence [30,31]. Addressing interpersonal problems early in marriage by teaching appropriate communications skills might help in the reduction of domestic violence. The focus of 4 out of 10 core sessions of our intervention is on communications skills (knowing and appreciating each other; listening and understanding each other, communicating respectfully and positively, and seeking and valuing each other’s opinion).

Among stresses of parenthood, we found that the pressures of conceiving an off-spring (preferably male) immediately after marriage without time for the couple to adjust to the transition and prepare themselves for the responsibilities was both stressful, and potentially harmful for the new born. This problem has been identified in other studies [32–35] and is particularly more profound in the South Asian culture [34,36]. Studies show that lack of knowledge and awareness about sexual and reproductive health compounds the issue, especially in matters of family planning and birth spacing [37]. The combination of health education and training in negotiation about such issues between family members might assist young couples be better prepared for becoming parents. The focus of three out of 10 core sessions was on helping the young couple deal with the stresses of parenthood at each stage (pre-pregnancy, delivery and post-pregnancy).

Our qualitative findings showed that interpersonal problems and stresses of parenthood had a profound impact on psychosocial well-being, especially of young women. There is a large body of evidence showing that the rates of anxiety and depression in women of reproductive age is high [38], especially in Pakistan [39]. These and other quantitative studies have also indicated similar stresses to contribute to the high rates of anxiety and depression [38]. Maternal depression has a negative impact on child growth, cognitive development and other health outcomes [40,41]. It is therefore important to address psychosocial distress at the same time as life-skills and health-education. Our intervention has integrated components from evidence-based strategies that have been successfully used in the study area in a number of studies [25,41]. It would be important to include interventional strategies that have a role in preventing as well as treating symptoms of depression in young couples, especially women. We were able to integrate CBT-based techniques into the life-skills intervention [26], and envisage this would have a synergistic effect in improving parental and child outcomes.

Our study had some limitations. We purposively selected willing young couples for interview. Those who did not engage with the process might have divergent views which would not be captured. The participants were all from one rural District of Punjab, and their views might not be representative of the entire population. All the interviewers were educated young women and this may have introduced some unconscious bias in the responses elicited, despite the lead investigator (NA) have frequent discussions with the interviewers to address this positionality.

We recognise that our study does not establish the acceptability or effectiveness of the intervention. Such questions would be answered in a pilot and feasibility study followed by a randomised controlled trial. However, the Medical Research Council’s framework for design and evaluation of complex interventions [42] requires developmental research as a necessary first step before embarking on pilot and definitive trials. This research is necessary to clarify the outcomes being aimed for, the proposed mechanism through which change would be affected, and establishing a coherent theoretical basis for the intervention. The intervention needs to be described fully so that it can be implemented properly for the purposes of evaluation, and replicated by others. Following these guidelines, we are currently undertaking a feasibility study of the intervention to establish its feasibility and plan for a larger definitive trial in the future. We envisage this will begin to fill the gap for such an intervention in LMICs.

## Data Availability

The dataset generated and analysed during the current study are available from the corresponding author on reasonable request.

## Paper context

Like many low resource settings, in Pakistan, there is a five-fold increase in prevalence rates of depression following marriage and pregnancy in younger women, possibly related to a rapid role transition. Currently, there is no intervention that equips young married couples with skills to negotiate these life transitions in low resource settings. This paper describes the development of Preparing for Parenthood program- a Life Skills Training intervention for young married couples that could potentially address this gap.
